# Simple and cost-effective liquid chromatography-mass spectrometry method to measure dabrafenib quantitatively and six metabolites semi-quantitatively in human plasma

**DOI:** 10.1007/s00216-017-0316-8

**Published:** 2017-04-20

**Authors:** Svante Vikingsson, Jan-Olof Dahlberg, Johan Hansson, Veronica Höiom, Henrik Gréen

**Affiliations:** 1 Division of Drug Research, Department of Medical and Health Sciences, Faculty of Health Sciences, Linköping University, 581 85 Linköping, Sweden; 2 Department of Oncology-Pathology, Karolinska Institutet, Karolinska University Hospital Solna, 171 64 Stockholm, Sweden; 3 Department of Forensic Genetics and Forensic Toxicology, National Board of Forensic Medicine, Artillerigatan 12, 587 58 Linköping, Sweden

**Keywords:** Bioanalytical methods, Biological samples, Drug monitoring/drug screening, HPLC, Mass spectrometry/ICP-MS

## Abstract

Dabrafenib is an inhibitor of BRAF V600E used for treating metastatic melanoma but a majority of patients experience adverse effects. Methods to measure the levels of dabrafenib and major metabolites during treatment are needed to allow development of individualized dosing strategies to reduce the burden of such adverse events. In this study, an LC-MS/MS method capable of measuring dabrafenib quantitatively and six metabolites semi-quantitatively is presented. The method is fully validated with regard to dabrafenib in human plasma in the range 5–5000 ng/mL. The analytes were separated on a C18 column after protein precipitation and detected in positive electrospray ionization mode using a Xevo TQ triple quadrupole mass spectrometer. As no commercial reference standards are available, the calibration curve of dabrafenib was used for semi-quantification of dabrafenib metabolites. Compared to earlier methods the presented method represents a simpler and more cost-effective approach suitable for clinical studies.

**Electronic supplementary material:**

The online version of this article (doi:10.1007/s00216-017-0316-8) contains supplementary material, which is available to authorized users.

**Graphical abstract:**
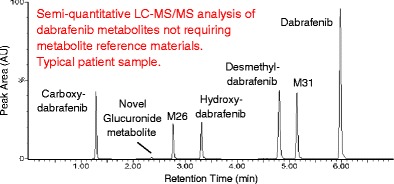
Combined multi reaction monitoring transitions of dabrafenib and metabolites in a typical case sample.

## Introduction

Approximately half of all cutaneous melanomas display somatic mutations in the BRAF gene and in 90% of those the mutation is a single amino acid substitution from valine to glutamic acid at codon 600 in exon 15 (BRAF V600E) [1]. The mutated enzyme activates the mitogen-activated protein kinases (MAPK) pathway leading to cell proliferation [2].

Dabrafenib (GSK2118436), registered as Tafinlar®, is an inhibitor of BRAF V600E approved for treatment of non-resectable stage III or stage IV metastatic melanoma carrying the mutation. In a phase III trial, 53% of patients experienced adverse events of grade 2 or greater including squamous cell carcinoma (SCC), pyrexia, fatigue, headache, arthralgia, and different cutaneous adverse drug reactions [3]. In order to limit the effects of these adverse drug reactions through individualized dosing of dabrafenib, it is important to study the pharmacokinetics of the drug.

After oral absorption dabrafenib is converted into hydroxy-dabrafenib mainly by cytochrome P450 2C8 (CYP2C8) and CYP3A4. Hydroxy-dabrafenib is further metabolized into carboxy-dabrafenib by CYP3A4, which is non-enzymatically converted into desmethyl-dabrafenib [4, 5]. Together, these metabolites constituted 76% of the circulating radioactivity in humans after administration of 14C-labeled dabrafenib. Minor metabolites include a glucuronide of hydroxyl-dabrafenib and further oxidations of desmethyl-dabrafenib [4]. The pharmacokinetics of dabrafenib exhibits a great deal of inter-patient variability. A 73% inter-patient coefficient of variation (CV) in maximum concentration at steady state was observed [6]. The metabolism of dabrafenib is rapid with a half-life of 4.8 h [7]. Even though dabrafenib is given twice daily, Falchook et al. [8] showed that the plasma concentration of dabrafenib and hydroxy-dabrafenib vary 16- and 7-fold, respectively, during a 12-h period after a 150-mg dose of dabrafenib at steady state (day 15). However, the concentrations of carboxy-dabrafenib and desmethyl-dabrafenib were more stable (around 1.5-fold variability).

Predictions of clinical effect and toxicity is further complicated by the fact that hydroxyl-dabrafenib and desmethyl-dabrafenib show activity on BRAFV600E with IC50 values approximately 3- and 2-fold higher than dabrafenib, respectively [9]. Given observed plasma levels, it is likely that these metabolites contribute significantly to the clinical effect and/or toxicity. It is therefore of importance to evaluate the impact of inter-patient variability in metabolite plasma levels on clinical efficacy and risk of adverse events and thus a need for analytical methods providing information on the plasma levels of dabrafenib metabolites.

Two methods to measure dabrafenib in plasma have previously been described [4, 10]. The method by Sparidans et al. [10] used protein precipitation followed by dilution coupled to reversed-phase liquid chromatography-tandem mass spectrometry (LC-MS/MS) to analyze dabrafenib in mouse plasma using an analogue of vemurafenib, PLX4720, as the internal standard. The method does not detect any metabolites and was not designed for human plasma. The method by Bershas et al. [4] used two separate liquid-liquid extractions to analyze dabrafenib and three metabolites (hydroxyl-, carboxy-, and desmethyl-dabrafenib) in human plasma. The method used isotopically labeled internal standards for all analytes. Although the method allows the possibility of measuring dabrafenib metabolites in plasma the two separate sample preparations are impractical and the lack of commercially available metabolite reference standards makes it expensive to implement in the laboratory.

A more cost-efficient approach successfully used previously by us (research groups of Vikingsson and Gréen) for BRAF inhibitor vemurafenib [11] and EGFR-inhibitor erlotinib [12] is to use human liver microsomes (HLMs) and patient samples to identify metabolites and use the parent drug as the calibrator.

The aim of this study was to develop and validate a simple and affordable LC-MS/MS method to measure dabrafenib and all major metabolites for potential use in clinical trials.

## Material and methods

### Chemicals and reagents

Dabrafenib (purity >99%) was obtained from Selleck Chemicals (Munich, Germany). Internal standard Erlotinib-d6 was acquired from Toronto Research Chemicals Inc. (North York, ON, Canada). Ammonium acetate and dimethyl sulfoxide (DMSO) of analytical grade or higher were obtained from Sigma Aldrich Sweden AB (Solna, Sweden). Acetonitrile hypergrade for LC-MS LiChrosolv from VWR (Stockholm, Sweden). Purified water was prepared in the laboratory using a Milli-Q Gradient purchased from Millipore AB (Solna, Sweden).

Seven blood samples were obtained between 14 and 17 h after last dose from four different patients with disseminated melanoma after 1, 2, or 3 months of treatment with dabrafenib at the full recommended dose of 150 mg BID orally. After 1 month, patients are expected to have reached steady-state levels of dabrafenib and metabolites (the half-life of dabrafenib and metabolites was reported to be 4.8 h and less than 21 h, respectively [7]). Plasma was collected after centrifugation, immediately frozen, and stored at −80 °C until analysis. The study protocol was approved by the Ethical Review Board in Stockholm (DNR 2011/1980-31/1) and written informed consent was obtained from patients prior to study entry. Drug-free ethylenediaminetetraacetic acid (EDTA) plasma was donated from six volunteers (three males and three females) with an average age of 26 (22–30) years.

Human liver microsomes (pool of 200 donors), NADPH regenerating system solution A (26 nM NADP+, 66 mM glucose-6-phosphate, and 66 mM MgCl_2_), NADPH regenerating system solution B (40 U/mL glucose-6-phosphate dehydrogenase), UGT reaction mixture solution A (25 mM uridine-5′-diphospho-glucoronic acid (UGDPA)), and UGT mixture solution B (250 mM Tris-HCl, 40 mM MgCl_2_, and 0.125 mg/mL alamethicin, pH 7.5) were purchased from BD Bioscience (Stockholm, Sweden).

### Chromatographic conditions

The chromatographic separation was performed using an Acquity ultraperformance liquid chromatography (UPLC) system consisting of column manager, sample manager, and binary solvent manager, all from Waters (Sollentuna, Sweden). Three microliters of sample was separated on an Acquity UPLC BEH C18 column (100 × 2.1 mm, 1.7 μm) with a BEH VanGuard C18 guard Column (5 × 2.1 mm, 1.7 μm) maintained at 55 °C. Five millimolars ammonium acetate and acetonitrile was used as mobile phases with a flow rate pf 0.65 mL/min. The analytes were eluted with a linear gradient from 20 to 45% acetonitrile for the first 6 min (0–6 min), before returning to initial conditions of 20% acetonitrile to re-equilibrate the last minute (6–7 min). Due to the short equilibration time, the first two injections of a sequence should be ignored.

MS/MS detection was performed using a Xevo TQ triple quadrupole mass spectrometer operated in positive electrospray ionization (ESI) mode, also from Waters. The source temperature was 150 °C, desolvation temperature 600 °C, cone gas flow 24 L/h, desolvation gas flow 1000 L/h, and collision gas flow 0.14 mL/min (argon). The analytes were monitored in multireaction monitoring mode (MRM). Specific parameters are given in Table 1.

**Table 1:** Details of mass spectrometric detection

Compound	Retention time (min)	Measurement window (min)	Precursor ion (*m*/*z*)	Cone voltage (V)	Product ion (*m*/*z*)	Collision energy (eV)	Dwell time (ms)
Dabrafenib	6.01	5.7–7.0	520.1	48	307.2	36	35
					277.0	70	35
Erlotinib-d6	4.62	3.7–5.7	400.2	44	287.2	30	35
					339.2	22	35
Carboxy-Dab	1.32	1.2–1.9	550.1	35	329.1	35	35
					314.2	55	35
Gluc met (novel)	2.18	2.1–2.5	696.1	40	291.1	73	21
					328.3	60	21
M26	2.82	2.1–3.2	522.2	47	309.1	40	21
					291.1	46	21
Hydroxy-Dab	3.36	3.2–3.7	536.2	50	293.1	48	75
					323.1	39	75
Desmethyl-Dab	4.84	4.4–5.0	506.2	46	293.2	40	35
					309.1	37	35
M31	5.19	5.0–5.7	504.1	40	291.1	43	35
					307.1	32	35

*Dab* dabrafenib, *Gluc met* glucuronidated metabolite

### Sample preparation

Fifty microliters aliquots of plasma was thawed and precipitated with 200 μL of internal standard mixture containing erlotinib-d6 (200 ng/mL) in acetonitrile. Following vortex mixing, the samples were centrifuged (17530 RCF, 5 min, 4 °C). The supernatant (150 μL) was mixed with 150 μL water and transferred to a 0.7-mL 96-well plate.

### Stock solutions, calibration, and controls

Dabrafenib was dissolved in DMSO to a concentration of 1 mg/mL. Internal standard erlotinib-d6 was dissolved in DMSO to a concentration of 1.9 mg/mL. Calibration and quality control (QC) working stock solutions were prepared from the stock solutions by dilution in an acetonitrile/water (50:50, *v*/*v*) mixture.

Calibration standards for dabrafenib were prepared at seven levels in the concentration range 5–5000 ng/mL (5-15-60-250-1000-3750-5000) by diluting the corresponding working stock solutions 1:10 with drug-free plasma. The upper limit of quantification (ULOQ) was selected based on the highest expected dabrafenib concentration, and the lower limit of quantification (LLOQ) was selected based on observed carry-over from the ULOQ. LOD was not determined as LLOQ was limited by the carry-over. QC samples were prepared separately from the calibration standards at four levels (LLOQ-low-medium-high, 5-15-250-3750 ng/ml).

Due to the lack of reference materials, the six metabolites were semi-quantitatively determined using the calibration curve for dabrafenib. The bias caused by, among other things, differences in recovery and ionization efficiency was not corrected for. This bias is indicated by the addition of an asterisk to the units of metabolite concentrations.

### Human liver microsome incubations

Dabrafenib was incubated at 10 μM for 120 min. For an incubation volume of 1 mL, 50 μL NADPH regenerating system solution A, 10 μL NADPH regenerating system solution B, 200 μL UGT mixture solution A, 80 μL UGT mixture solution B, and 25 μL HLM (0.5 mg protein) were diluted to 995 μL in water and preheated to 37 °C in a water bath. The reaction was started by addition of 5 μL dabrafenib stock solution (5 mM in DMSO) and terminated by addition of 150 μL of incubate to four volumes of ice-cold acetonitrile. After centrifugation, the supernatant was evaporated under nitrogen and reconstituted in mobile phase. A negative control without dabrafenib was used as well as a degradation control without HLM.

### Metabolite identification

Metabolites previously reported by Bershas et al. [4] were primarily identified after incubations with HLM. Single ion monitoring (SIM) was used to identify the molecular ion and multireaction monitoring (MRM) transitions were established based on those reported by Bershas et al. [4]. The naming convention presented by Bershas et al. [4] was maintained. The MRM transitions were optimized in a stepwise fashion with regard to Q1 and Q3 mass, cone voltage, and collision energy using intra-run comparisons of different settings. Only metabolites identified in patient plasma were included in the final method. For the optimized transitions, see Table 1.

Previously unknown metabolites were identified by full scan of the HLM extract in the 200–800 *m*/*z* range (segmented over several runs to increase sensitivity). To be considered a metabolite of interest it had to be present in patient plasma but not in HLM-negative control nor blank plasma. The structures of unknown metabolites were assigned based on obtained product ion spectra, which was also the basis for MRM transitions. MRM transitions were further optimized as detailed above for previously reported metabolites.

### Method validation

For dabrafenib the method was validated according to international guidelines from the Food and Drug Administration (FDA) [13] and the European Medicines Agency (EMA) [14] for bioanalytical methods.

The validity of the quadratic calibration model (weighted 1/*x* ^2^) was established by back-calculating concentrations for calibration standards from six independent runs. Carry-over was evaluated by analyzing drug-free plasma after the highest calibration standard (*n* = 3). Intra-batch (*n* = 6) and inter-batch (*n* = 6) accuracy and precision were determined at all four QC levels. Selectivity was determined by analysis of drug-free plasma obtained from six individuals.

The matrix factor at low and high QC levels were evaluated in six individuals and determined as the area ratio of dabrafenib between spiked drug-free plasma from six individuals and dabrafenib spiked in 40% acetonitrile (*n* = 3). The factor was normalized by the corresponding internal standard matrix factor [13, 14].

Recovery at QC low and QC high was evaluated by spiking drug-free plasma from six individuals and determined as the area ratio between a sample spiked before extraction and a sample spiked after extraction adjusted for internal standard recovery.

Stability was evaluated at the low and high QC levels. Processed sample stability by re-injection of calibration and QC samples after 24 h at 5 °C. Freeze-thaw stability (*n* = 3) was determined for three freeze-thaw cycles at −80 °C. Short-term stability of dabrafenib was investigated in whole blood and plasma using spiked drug-free plasma (*n* = 2) stored at room temperature or 5 °C for 0, 24, 48, and 72 h, respectively. Long-term stability of dabrafenib (QC low, QC middle, and QC high; *n* = 3) was determined for a sample stored at −80 °C for 16 months. Accuracy within ±15% of the nominal value was required to consider dabrafenib stable under all conditions.

As no reference standards were available, a full validation of the metabolite measurements was not possible. Instead, the specificity was validated analyzing blank samples (*n* = 6) and the repeatability was determined by duplicate analysis (inter-day) of patient samples (*n* = 7). The latter is not supported by the validation guidelines used [13, 14].

## Results

**Fig. 1:**
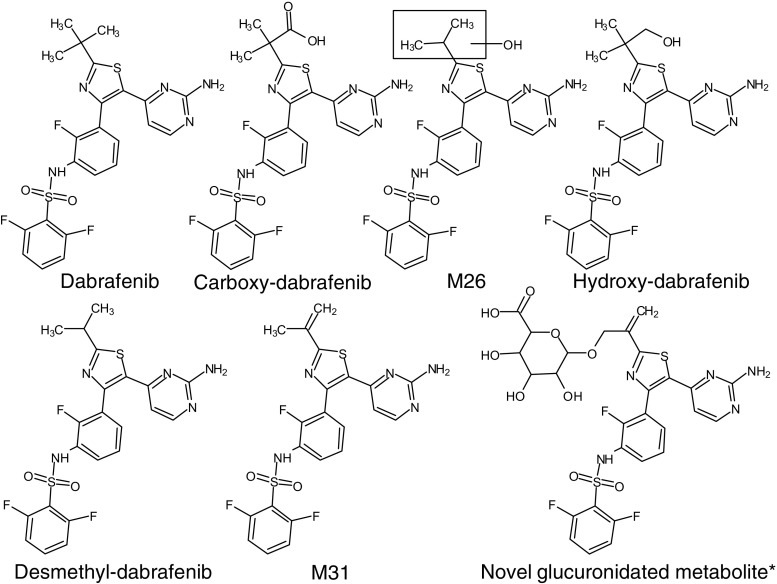
Structure suggestions for identified metabolites. The *asterisk* denotes that several other structures are possible

In this project, a method was developed to measure dabrafenib and six metabolites, including a glucuronidated metabolite previously not described. Names of metabolites, including M26 and M31, were selected to correspond to those used by Bershas et al. [4]. Possible structures are given in Fig. 1. The novel glucuronidated metabolite is suggested to be the conjugate of metabolite M31 based on the mass of the molecule, the fragment 291 also observed in M31, and the fact that the isopropyl moiety seems to be a prevalent site of hydroxylation. However, several other structures are possible.

**Fig. 2:**
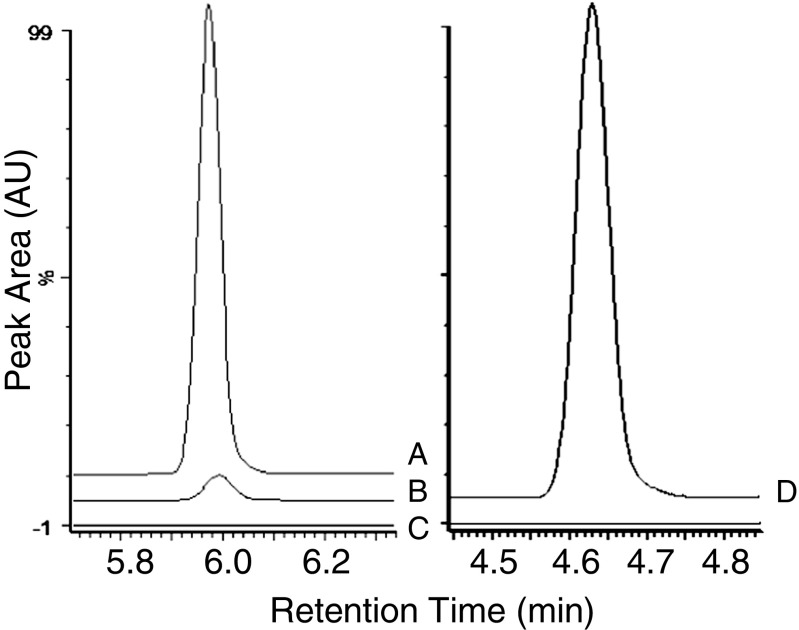
Chromatograms of dabrafenib in lowest patient sample (*A*, 75 ng/mL) and at LLOQ (*B*, 5 ng/mL) compared to blank (*C*) and the internal standard D6-erlotinib (*D*)

**Fig. 3:**
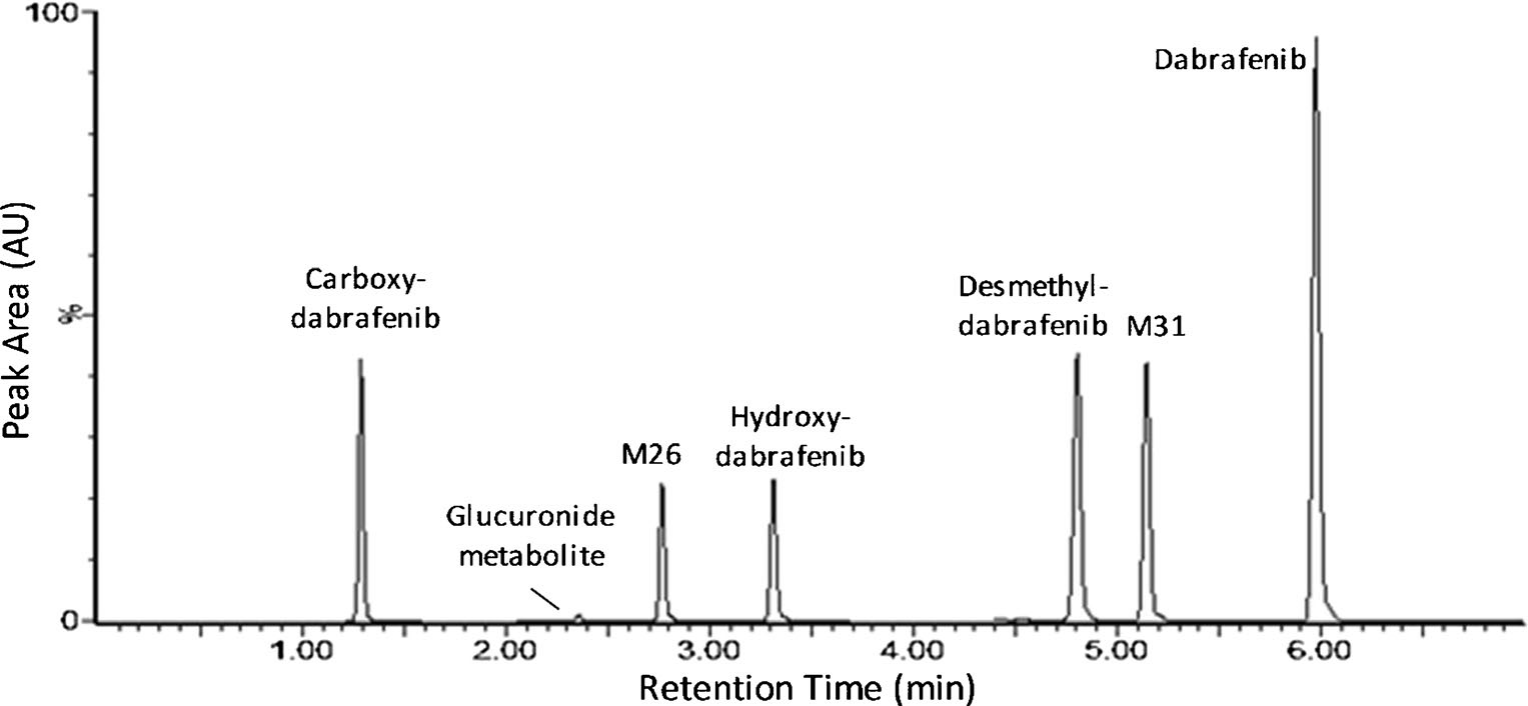
Typical chromatogram of patient sample. Carboxy-dabrafenib 400 ng/mL*, glucuronidated metabolite 32 ng/mL*, M26 230 ng/mL*, hydroxyl-dabrafenib 280 ng/mL*, desmethyl-dabrafenib 660 ng/mL*, M31 550 ng/mL*, and dabrafenib 1400 ng/mL. *Semi-quantification based on the calibration curve of dabrafenib

Representative chromatograms of dabrafenib at LLOQ are shown in Fig. 2. Chromatograms of the metabolites are shown in Fig. 3 and estimated concentration ranges for each metabolite (based on dabrafenib calibration) are found in Table 3.

### Structure assignment of novel metabolite

The novel glucuronide metabolite was identified based on the presence of peaks *m*/*z* 291 and *m*/*z* 328 in the product ion spectra, see Electronic Supplementary Material (ESM) Fig. S1. Even though the mechanism of formation is unclear, the fragment *m*/*z* 291 was reported by Bershas et al. [4] for metabolites M26 and M31, both of which are modified solely at the isobutyl group. This is also to some extent supported by *m*/*z* 328, formed by the loss of the glucuronide and the difluorobenzenesulfonamide moiety.

### Method validation

The calibration model was valid with as the average back-calculated concentrations were 97–103% for all calibration standards (see ESM Table S1).

The carry-over was on average 0.02% (corresponding to 14% of LLOQ) and 0.06% for dabrafenib and D6-erlotinib, respectively (see ESM Table S2).

Intra- and inter-batch accuracy was within 86–95 and 89–91% for all QC levels, respectively. Intra- and inter-batch imprecision (CV) was below 7 and 11% for all QC levels, respectively. Details can be seen in Table 2. An accuracy of 86% and a precision of 7% validated the suitability of the selected LLOQ.

**Table 2:** Accuracy and precision

	Intra-batch (*n* = 6)		Inter-batch (*n* = 6)	
QC level	Accuracy (%)	Precision (%)	Accuracy (%)	Precision (%)
LLOQ^a^	86	7	89	9
Low	88	7	91	8
Medium	95	3	91	11
High	92	2	91	8

^a^ *n* = 5

No interfering peaks for dabrafenib or any of the metabolites were identified in blank plasma (*n* = 6). A matrix factor of 2.06 and 1.28 was observed at low and high QC levels. It was stable with a CV of no more than 2% between different sources of plasma (*n* = 6) (see ESM Table S3). Recovery at low and high QC levels for dabrafenib and the internal standard was between 104 and 115%; IS-corrected recovery for dabrafenib was 110 and 96% at the low and high QC levels, respectively (see ESM Table S4).

Processed samples were stable for 24 h at 5 °C. Dabrafenib spiked in whole blood or plasma was stable for 72 h at room temperature and at 5 °C. In the plasma, dabrafenib was also stable for three freeze-thaw cycles and during 16 months of storage at −80 °C (see ESM Table S5).

## Discussion

The presented method was not developed as a TDM method, but to meet the need of our clinical studies, a method capable of correlating patient plasma levels with clinical outcomes such as efficacy and adverse events. As some metabolites have been shown to be active [9], it was important to include as many of the metabolites as possible. However, a method similar to that presented by Bershas et al. [4] requires substantial resources, which were not available.

A more cost-efficient approach was therefore selected in which the metabolites were semi-quantified using the dabrafenib calibration curve eliminating the need for custom synthesis reference materials. It did however introduce a bias that is unknown and potentially substantial. That said, the bias should be similar in all samples meaning that even though the true concentrations of the metabolites cannot be determined with any accuracy, the concentration in a given sample relative to other samples can. Hence, metabolite plasma levels can be correlated to i.e. adverse events. Should the plasma levels of any given metabolite correlate strongly with a particular adverse event that metabolite could be synthesized and easily added to the method. As the patient samples appear to be stable at −80 °C re-analysis providing accurate concentrations would then be possible.

As sensitivity was not a major issue and a blood sample provides more plasma than needed, the main concern when developing the method was to make the sample preparation cost-efficient. Protein precipitation was an obvious choice as it is rapid and requires a minimum of consumables. Protein precipitation was also used by Sparidans et al. [10] but that method was limited to dabrafenib and did not include any metabolites.

Given recent developments in treatment practice, dabrafenib is frequently administered together with trametinib. Although not included in the presented method, it has been shown that trametinib can be analyzed together with dabrafenib on a C18 column using ammonium acetate-based mobile phases [15, 16]. This indicates that trametinib and possible trametinib metabolites probably could be included into the presented method using a modified gradient.

In the method, six metabolites were included as seen in Table 1 and Fig. 3. Five of them were previously reported by Bershas et al. [4], which came as no surprise given the thoroughness of that excellent study. For these metabolites, the identity was ensured by verifying the presence of the four or five characteristic fragment ions provided by Bershas et al. [4]. For the novel metabolite, the identity was inferred by examining the product ion spectra (ESM Fig. S1) and even though the information was limited, the fragments identified the metabolite as a hydroxyl glucuronide of M31.

The described method was successfully validated with regard to dabrafenib. It comprises of a simple protein precipitation allowing all metabolites to be included in the same sample preparation. A substantial matrix factor was observed for dabrafenib but as this factor was highly reproducible between different matrices and as the calibration standards are prepared in the plasma, it will not significantly affect the precision or the accuracy of the method.

In general, the bias of the metabolite concentrations appeared to be reasonably small. Falchook et al. [8] reported similar metabolite concentrations as seen in Table 3 with the exception of carboxy-dabrafenib where they reported an 8-fold higher concentration than found in this study. As carboxy-dabrafenib is a carboxylic acid and the chromatography is run at neutral pH, it is likely to mainly be present as negative ions, which are hard to ionize in positive ESI mode. Thus, carboxy-dabrafenib is expected to ionize poorly in the presented method compared to dabrafenib, which was used for quantification, providing a plausible explanation for the differences in observed concentrations compared to Falchook et al. [8].

**Table 3:** Plasma concentrations

Compound	Median C (ng/ml)	Range	Diff
Dabrafenib	400	(75–1400)	19-fold
Carboxy-Dab	450^a^	(230–690)	3-fold
Gluc met	38^a^	(22–65)^b^	3-fold
M26	120^a^	(59–230)	4-fold
Hydroxy-Dab	74^a^	(19–278)	15-fold
Desmethyl-Dab	400^a^	(170–660)	4-fold
M31	400^a^	(160–590)	4-fold

*C* plasma concentration, *Dab* dabrafenib, *Diff* difference between the highest and lowest concentrations, *Gluc met* glucuronidated metabolite

^a^Semi-quantification based on the calibration curve of dabrafenib

^b^ *N* = 6 due to technical reasons

That said, the method is suitable to compare metabolite levels between patients in the ongoing clinical studies and identify metabolites correlated to treatment efficacy, adverse events, or genetic variability. If any such metabolites are identified, they can be synthesized and validated without changing the methodology used.

Steady-state levels of dabrafenib and metabolites showed considerable inter-patient variability although all patients were receiving the full-prescribed dose of dabrafenib (Table 3). This was in line with earlier observations by Suttle et al. [6] (73% CV in maximum concentration at steady state) and indicates that the inter-patient variability of dabrafenib is even higher than observed for vemurafenib (30–60%) 1[17].

Also, contrary to vemurafenib [11], a substantial amount of metabolites are present in the plasma at steady state, as can be seen in Fig. 3. A significant inter-patient variability in metabolite plasma levels was observed, especially for hydroxy-dabrafenib as seen in Table 3.

Substantial inter-patient variability in dabrafenib pharmacokinetics indicates a risk of highly variable plasma levels of dabrafenib and metabolites on standardized therapy. High plasma levels could possibly explain some of the observed adverse effects such as pyrexia and squamous cell carcinoma. On the other, low plasma levels could possibly induce resistance, as shown for other agents in vitro [18]. Together, these findings warrant further research into therapeutic drug monitoring and the importance of inter-patient pharmacokinetic variability of dabrafenib and its metabolites.

## Conclusions

In conclusion, a method capable of measuring dabrafenib and six metabolites is presented. The method is simpler and more cost-effective than previously published methods to measure metabolites. The method was fully validated with regard to dabrafenib and well suited for clinical studies.

## Electronic supplementary material

ESM 1 (PDF 170 kb)

## References

[1] Ascierto PA, Kirkwood JM, Grob JJ, Simeone E, Grimaldi AM, Maio M, Palmieri G, Testori A, Marincola FM, Mozzillo N (2012). The role of BRAF V600 mutation in melanoma. J Transl Med.

[2] Dhillon AS, Hagan S, Rath O, Kolch W (2007). MAP kinase signalling pathways in cancer. Oncogene.

[3] Hauschild A, Grob JJ, Demidov LV, Jouary T, Gutzmer R, Millward M, Rutkowski P, Blank CU, Miller WH, Kaempgen E, Martin-Algarra S, Karaszewska B, Mauch C, Chiarion-Sileni V, Martin AM, Swann S, Haney P, Mirakhur B, Guckert ME, Goodman V, Chapman PB (2012). Dabrafenib in BRAF-mutated metastatic melanoma: a multicentre, open-label, phase 3 randomised controlled trial. Lancet.

[4] Bershas DA, Ouellet D, Mamaril-Fishman DB, Nebot N, Carson SW, Blackman SC, Morrison RA, Adams JL, Jurusik KE, Knecht DM, Gorycki PD, Richards-Peterson LE (2013). Metabolism and disposition of oral dabrafenib in cancer patients: proposed participation of aryl nitrogen in carbon-carbon bond cleavage via decarboxylation following enzymatic oxidation. Drug Metab Dispos.

[5] Lawrence SK, Nguyen D, Bowen C, Richards-Peterson L, Skordos KW (2014). The metabolic drug-drug interaction profile of dabrafenib: in vitro investigations and quantitative extrapolation of the P450-mediated DDI risk. Drug Metab Dispos.

[6] Suttle AB, Grossmann KF, Ouellet D, Richards-Peterson LE, Aktan G, Gordon MS, LoRusso PM, Infante JR, Sharma S, Kendra K, Patel M, Pant S, Arkenau HT, Middleton MR, Blackman SC, Botbyl J, Carson SW (2015). Assessment of the drug interaction potential and single- and repeat-dose pharmacokinetics of the BRAF inhibitor dabrafenib. J Clin Pharmacol.

[7] Denton CL, Minthorn E, Carson SW, Young GC, Richards-Peterson LE, Botbyl J, Han C, Morrison RA, Blackman SC, Ouellet D (2013). Concomitant oral and intravenous pharmacokinetics of dabrafenib, a BRAF inhibitor, in patients with BRAF V600 mutation-positive solid tumors. J Clin Pharmacol.

[8] Falchook GS, Long GV, Kurzrock R, Kim KB, Arkenau HT, Brown MP, Hamid O, Infante JR, Millward M, Pavlick A, Chin MT, O'Day SJ, Blackman SC, Curtis CM, Lebowitz P, Ma B, Ouellet D, Kefford RF (2014). Dose selection, pharmacokinetics, and pharmacodynamics of BRAF inhibitor dabrafenib (GSK2118436). Clin Cancer Res.

[9] FDA (2013a) Clinical Pharmacology and Biopharmaceuticals review(s) application number 202806Orig1s000. U.S. Food and Drug Administration, Center for Drug Evaluation and Research. http://www.accessdata.fda.gov/drugsatfda_docs/nda/2013/202806Orig1s000ClinPharmR.pdf. Accessed 11–2-2017 2017.

[10] Sparidans RW, Durmus S, Schinkel AH, Schellens JH, Beijnen JH (2013). Liquid chromatography-tandem mass spectrometric assay for the mutated BRAF inhibitor dabrafenib in mouse plasma. J Chromatogr B Analyt Technol Biomed Life Sci.

[11] Vikingsson S, Stromqvist M, Svedberg A, Hansson J, Hoiom V, Green H (2016). Novel rapid liquid chromatography tandem mass pectrometry method for vemurafenib and metabolites in human plasma, including metabolite concentrations at steady state. Biomed Chromatogr.

[12] Svedberg A, Green H, Vikstrom A, Lundeberg J, Vikingsson S (2015). A validated liquid chromatography tandem mass spectrometry method for quantification of erlotinib, OSI-420 and didesmethyl erlotinib and semi-quantification of erlotinib metabolites in human plasma. J Pharm Biomed Anal.

[13] FDA (2013b) Guidance for industry bioanalytical method validation, draft guidance U.S. Food and Drug Administration, Center for Drug Evaluation and Research. http://www.fda.gov/downloads/drugs/guidancecomplianceregulatoryinformation/guidances/ucm368107.pdf Accessed 28–1-2015.

[14] EMA (2011) Guideline on bioanalytical method validation European Medicines Agency (EMA), Committee for Medicinal Products for Human Use (CHMP). http://www.ema.europa.eu/docs/en_GB/document_library/Scientific_guideline/2011/08/WC500109686.pdf. Accessed 28–1-2015.

[15] Huynh HH, Pressiat C, Sauvageon H, Madelaine I, Maslanka P, Lebbe C, Thieblemont C, Goldwirt L, Mourah S (2017). Development and validation of a simultaneous quantification method of 14 tyrosine kinase inhibitors in human plasma using LC-MS/MS. Ther Drug Monit.

[16] Nijenhuis CM, Haverkate H, Rosing H, Schellens JH, Beijnen JH (2016). Simultaneous quantification of dabrafenib and trametinib in human plasma using high-performance liquid chromatography-tandem mass spectrometry. J Pharm Biomed Anal.

[17] Grippo JF, Zhang W, Heinzmann D, Yang KH, Wong J, Joe AK, Munster P, Sarapa N, Daud A (2014). A phase I, randomized, open-label study of the multiple-dose pharmacokinetics of vemurafenib in patients with BRAF V600E mutation-positive metastatic melanoma. Cancer Chemother Pharmacol.

[18] Liu WM, Oakley PR, Joel SP (2002). Exposure to low concentrations of etoposide reduces the apoptotic capability of leukaemic cell lines. Leukemia.

