# Supplementary material for: Simple and cost-effective liquid chromatography-mass spectrometry method to measure dabrafenib quantitatively and six metabolites semi-quantitatively in human plasma
Source: Anal Bioanal Chem. 2017 Apr 20;409(15):3749–56. doi: 10.1007/s00216-017-0316-8 (PMC5427163; doi:10.1007/s00216-017-0316-8)
Supplement: Supplementary file 1 — (PDF 170 kb) [file 216_2017_316_MOESM1_ESM.pdf]

Analytical and Bioanalytical Chemistry

Electronic Supplementary Material

Simple and cost-effective liquid chromatography-mass spectrometry method to measure dabrafenib quantitatively and six metabolites semi-quantitatively in human plasma

Svante Vikingsson, Jan-Olof Dahlberg, Johan Hansson, Veronica Höiom Henrik Gréen

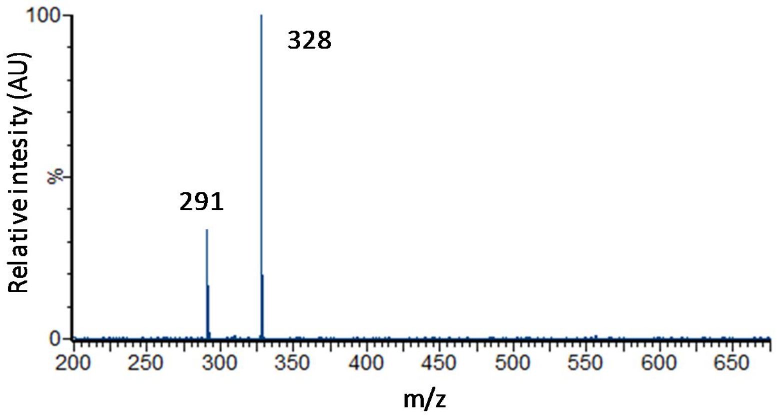

Fig. S1 Product ion spectra for novel glucuronide metabolite

Table S1 Validity of calibration model

Std	Nominal conc (ng/ml)	Back-calculated concentrations (ng/ml) Curve 1	Curve 2	Curve 3	Curve 4	Curve 5	Curve 6	Mean	CV
**1**	5	4,57	4,66	4,49	5,40	5,13	5,29	**98%**	**8%**
**2**	15	15,1	15,1	16,0	14,9	15,2	14,9	**101%**	**3%**
**3**	60	61,2	60,8	59,4	56,3	55,9	56,9	**97%**	**4%**
**4**	250	263	262	261	249	253	251	**103%**	**2%**
**5**	1000	1025	1018	1013	986	1025	999	**101%**	**2%**
**6**	3750	3550	3587	3591	3853	3652	3764	**98%**	**3%**
**7**	5000	5191	5158	5195	4905	5078	4988	**102%**	**2%**

Std, standard; conc, concentration; CV, coefficient of variation.

Table S2 Carry-over

	Curve 1	Curve 2	Curve 3	Average
**Area std 1**	2 588	2 629	1 830	**2 349**
**Area std 7**	1 884 430	2 312 788	1 648 605	**1 948 608**
**Area blank**	233	560	201	**331**
**IS area std 7**	578 975	628 523	624 899	**610 799**
**IS srea blank**	379	527	264	**390**
**Carry-over Dabrafenib**	0,012%	0,024%	0,012%	**0,02%**
**% std 1**	9,0%	21,3%	11,0%	**14%**
**Carry-over IS**	0,065%	0,084%	0,042%	**0,06%**

Std, standards; IS, internal standard

Table S3 Matrix factors

Samples spiked in final buffer

	IS Area			Dabrafenib area	
**Rep**	**Low QC**	**High QC**	**Rep**	**Low QC**	**High QC**
1	678 252	673 633	1	3 431	1 065 864
2	684 241	685 550	2	3 358	1 044 406
3	682 683	685 370	3	3 553	1 094 156
**Mean**	**681 725**	**681 518**	**Mean**	**3 447**	**1 068 142**
**CV**	**0%**	**1%**	**CV**	**3%**	**2%**

Samples spiked after extraction

	IS Area			Dabrafenib area	
**Rep**	**Low QC**	**High QC**	**Rep**	**Low QC**	**High QC**
1	649 323	707 519	1	6 690	1 373 240
2	674 242	709 066	2	7 041	1 399 080
3	664 810	714 220	3	6 914	1 436 380
4	683 566	710 942	4	7 124	1 411 395
5	680 335	718 877	5	7 102	1 484 703
6	678 800	733 682	6	7 198	1 492 474

Matrix factors*

	IS			Dabrafenib			Dabrafenib IS corrected	
**Rep**	**Low QC**	**High QC**	**Rep**	**Low QC**	**High QC**	**Rep**	**Low QC**	**High QC**
1	95%	104%	1	194%	129%	1	204%	124%
2	99%	104%	2	204%	131%	2	207%	126%
3	98%	105%	3	201%	134%	3	206%	128%
4	100%	104%	4	207%	132%	4	206%	127%
5	100%	105%	5	206%	139%	5	206%	132%
6	100%	108%	6	209%	140%	6	210%	130%
**Mean**	**99%**	**105%**	**Mean**	**203%**	**134%**	**Mean**	**206%**	**128%**
**Std dev**	**2%**	**1%**	**Std dev**	**5%**	**4%**	**Std dev**	**2%**	**3%**

* Is factors were calculated by dividing the area of samples spiked after extraction with the mean area in samples spiked in final buffer. Rep, replicate; IS, internal standard; CV, coefficient of variation.

Table S4 Recovery

Samples spiked before extraction

	IS Area			Dabrafenib area	
**Rep**	**Low QC**	**High QC**	**Rep**	**Low QC**	**High QC**
1	664 181	664 122	1	6 424	1 179 511
2	642 474	649 509	2	5 920	1 188 096
3	647 599	650 613	3	6 072	1 325 406
4	641 906	641 120	4	5 681	1 541 422
5	639 838	642 401	5	6 186	1 587 500
6	644 906	660 139	6	6 421	1 343 747
**Mean**	**646 817**	**651 317**	**Mean**	**6 117**	**1 360 947**
**CV**	**1%**	**1%**	**CV**	**5%**	**13%**

Samples spiked after extraction

	IS Area			Dabrafenib area	
**Rep**	**Low QC**	**High QC**	**Rep**	**Low QC**	**High QC**
1	649 323	707 519	1	6 690	1 373 240
2	674 242	709 066	2	7 041	1 399 080
3	664 810	714 220	3	6 914	1 436 380
4	683 566	710 942	4	7 124	1 411 395
5	680 335	718 877	5	7 102	1 484 703
6	678 800	733 682	6	7 198	1 492 474
**Mean**	**671 846**	**715 718**	**Mean**	**7 011**	**1 432 879**
**CV**	**2%**	**1%**	**CV**	**3%**	**3%**

Recovery

	**Low QC**	**High QC**
IS	104%	110%
Dabrafenib	115%	105%
Dabrafenib IS corrected	110%	96%

Rep, replicate; IS, internal standard; CV, coefficient of variation.

Table S5 Sample stability

Whole blood stability

	Low QC (ng/ml)*				High QC (ng/ml)*		
**Rep**	**0h**	**72 h RT**	**72 h 4°C**	**Rep**	**0h**	**72 h RT**	**72 h 4°C**
1	12,91	11,85	12,83	1	2 287	2 105	2 201
2	12,60	12,18	12,55	2	2 269	2 117	2 231
3	12,78			3	2 146		
**Mean**	**12,76**	**12,01**	**12,69**	**Mean**	**2 234**	**2 111**	**2 216**
**Rel**	**100%**	**94%**	**99%**	**Rel**	**100%**	**95%**	**99%**

Plasma stability

	Low QC (ng/ml)				High QC (ng/ml)		
**Rep**	**0h**	**72 h RT**	**72 h 4°C**	**Rep**	**0h**	**72 h RT**	**72 h 4°C**
**1**	14,587	14,111	16,324	**1**	4 073	3 900	4 157
**2**	15,219	14,979	16,182	**2**	4 023	4 033	4 235
**3**	14,814			**3**	3 726		
**Mean**	**14,87**	**14,55**	**16,25**	**Mean**	**3 941**	**3 966**	**4 196**
**Rel**	**100%**	**98%**	**109%**	**Rel**	**100%**	**101%**	**106%**

Processed sample stability

	Low QC (ng/ml)			High QC (ng/ml)	
	**0h**	**24 h 5°C**		**0h**	**24 h 5°C**
	15,10	13,21		3813	3382
**Rel**	**100%**	**87%**	**Rel**	**100%**	**89%**

Freeze-thaw stability in plasma

	Low QC (ng/ml)			High QC (ng/ml)	
**Rep**	**Before**	**After**	**Rep**	**Before**	**After**
1	16,30	16,05	1	4 504	4 410
2	15,62	15,31	2	4 491	4 002
3	15,94	15,65	3	4 258	4 365
**Mean**	**15,95**	**15,67**	**Mean**	**4 417**	**4 259**
**Rel**	**100%**	**98%**	**Rel**	**100%**	**96%**

Long term stability in plasma**

	Low QC (ng/ml)			High QC (ng/ml)	
**Rep**	**Before**	**After**	**Rep**	**Before**	**After**
1	15	14,90	1	3 750	3 881
2	15	14,83	2	3 750	3 983
3	15	15,55	3	3 750	4 052
**Mean**	**15,00**	**15,09**	**Mean**	**3 750**	**3 972**
**Rel**	**100%**	**101%**	**Rel**	**100%**	**106%**

* Due to an unknown amount of red blood cells in the whole blood the recovered concentration in plasma do not correspond to the QC levels used elsewhere. ** For long term stability the measured concentrations were compared to nominal concentrations of the QC levels. Rep, replicate; Rel; Concentration relative to initial sample concentration.
